# Equally Bad, Unevenly Distributed: Gender and the ‘Black Box’ of Student Employment

**DOI:** 10.1111/1468-4446.13210

**Published:** 2025-05-04

**Authors:** Mia Ruijie Zhong, Rachel Lara Cohen, Kim Allen, Kirsty Finn, Kate Hardy, Cassie Kill

**Affiliations:** ^1^ Leeds University Business School University of Leeds Leeds UK; ^2^ Department of Sociology and Criminology City St George's University of London London UK; ^3^ School of Sociology and Social Policy University of Leeds Leeds UK; ^4^ Manchester Institute of Education University of Manchester Manchester UK; ^5^ School of Education University of Sheffield Sheffield UK

**Keywords:** education, gender, student work, working‐life‐course, youth transitions

## Abstract

Students comprise approximately four per cent of the UK labour force and as much as 20% in some occupations and jobs. Yet students' work is typically seen as marginal, secondary both to their current learning and future working biographies. Public and media attention on ‘earning while learning’ (EwL) tends to focus on the negative impacts of paid work on education. Meanwhile students' actual working conditions, occupations and employment experiences have received limited attention and constitute something of a ‘black box’. We open that box by examining the paid work undertaken by full‐time students. Through analysis of a national data set, we examine patterns with respect to employment rates, pay, hours, and occupations, as well as how these are gendered. We find a small ‘studentness’ penalty—lower pay for students than non‐student workers of the same age. We also find small increases in the proportion currently engaged in paid work. Gender is identified as a key variable in shaping student employment rates, with women considerably more likely than men to work while studying. We find no evidence of a gender pay gap in EwL, but this is largely because most student workers are concentrated in two ‘integrated’ occupations, which we designate as ‘equally bad’ ‐ poorly paid but gender equitable. Older students are more likely to work in gender‐segregated occupations, with some indications of male and female gender pay advantages for gender‐dominant employment, suggesting a possible early incentive for occupational gender segregation. Given the gender disparity in student work, a core finding is that women disproportionately undertake this poor‐quality work. We argue that to address the under‐theorisation of EwL, student employment—including its gendering—requires greater attention and should be integrated into conceptualisations of a ‘working‐life‐course’.

## 1 Introduction

Young people in the UK—and globally—increasingly engage in paid employment while in school, further or higher education (Beerkens et al. 2011; Howieson et al. 2012; McKechnie et al. 2010; Taylor et al. 2020). This trend has been increasing over time (Callender 2008; Neves and Stephenson 2023). Not only are more students working while studying, but many work for longer hours (Neves and Stephenson 2023) and earnings from paid work now comprise over a quarter of average full‐time student income in England (NatCen Social Research & Institute for Employment Studies 2023).

The intersection of economic conditions and national educational policies render student work more necessary, particularly for those from lower socio‐economic backgrounds. Neoliberal education reforms in many countries have led to higher student fees and reductions in governmental financial support through loans and grants (Hordósy et al. 2018). Compounding this, the ongoing ‘cost‐of‐living crisis’ has squeezed students' everyday finances. Concerns abound that the increasing necessity of paid work has diverted students' attention from study, with detrimental impacts on educational outcomes and personal wellbeing (D. A. Jones 2022; Office for Students 2023).

Curiously, however, despite evidence that earning while learning (EwL) is widespread, and despite increasing concerns about the consequences of EwL, very little attention focused on students as a working labour force. While there is some variation in how ‘work’ ‐ both present and future—is incorporated into student identities across different national contexts (see Beerkens et al. 2011; Brooks et al. 2021), where students are acknowledged as workers, typically they are recognised as ‘future workers’ in the making (Brooks 2018). Student employment is therefore somewhat of a ‘black box’: little is known regarding types of work students engage in or how students' experiences of earning while learning may shape their identities and biographies as workers. This article begins to open that box by focusing on the paid work undertaken by full‐time students, asking what patterns exist with respect to employment rates, pay, hours and occupation and how these are gendered.

This article emerges from a large mixed‐methods project (‘L‐earning: rethinking young women’s working lives’) exploring young women's earliest experiences of work in England—including work undertaken whilst studying or ‘EwL’ ‐ and to trace the extent to which this prefigures later labour market outcomes, including the gendering of work. Drawing on data from the Annual Population Survey (APS), our analyses show consistent patterns of EwL over the past 2 decades, with students comprising a significant segment of the labour force in certain sectors. Crucially, women are more likely than men to work while studying, especially among school‐aged students. We found no evidence for overall gender pay gaps within the student labour force, but this is primarily explained by a large share of students in occupations that are equally, but poorly paid, with a large minority of students earning below national minimum wage rates *for their age* and the large majority earning below the full adult minimum wage level. In addition, we found a ‘studentness penalty’ for students aged 21–29, who were more likely than non‐students of a similar age to be in the worst paid work.

Finally, while we found that there was no overall gender pay gap between student workers, our results show that older students are more likely to work in gender‐segregated occupations, some of which offer pay advantages for gender‐dominant matching.

We use these original empirical findings to argue that EwL should be theorised as work equivalent in significance to any other and recognised as part of young people's work histories. The wider significance of such a framing is that it enables recognition of the role of EwL in potentially influencing trajectories, choices and gendered patterning in the labour market. It therefore contributes to richer theorisations of (gendered) patterns later in the working life course.

In what follows, we first outline the existing literature on student work and employment. We posit that three conceptual approaches have laid the foundations for beginning to understand EwL—youth transitions, employability and ‘anticipatory socialisation’. Second, in the methodology section, we outline our approach to analysis of Annual Population Survey data. Third, the results detail the five core findings: (1) relatively stable employment for a sizeable portion of student workers; (2) young female students are more likely to work than their male counterparts; (3) widespread low earnings with a small ‘studentness penalty’ for older students; (4) equally bad—gender equitable but poor—earnings; and, (5) sectoral concentration combined with growing occupational gendering among older students. We conclude by calling for more research on student employment that conceptualises it as part of the working‐life course and for greater support from policy makers including ensuring that student workers have and can exercise full employment rights.

## 2 Literature Review

### 2.1 Youth Transitions, Employability, and ‘Anticipatory Socialisation’: Theorising Earning While Learning (EwL)

Much discussion around young people, education and work is located within the well‐established field of ‘Youth Transitions’ research. This field has made valuable observations and criticisms of the metaphors of movement describing young people transitioning *from* education *into* work, whether this involves ‘pathways’ or ‘trajectories’ (Furlong 1997), ‘fast and slow lanes’ (Bynner et al. 2002) or more complex ‘yo‐yo’ movements (Biggart and Walther 2016). Yet, while there is broad consensus that young people's transitions have changed significantly over the last 50 years (see Goodwin and O’Connor 2005), the notion that education and work constitute two separate (and temporally‐ordered) spheres for young people remains. Yet, as we demonstrate, a high proportion of students are employed and more than 40% of working students remain in their job at least a year, signifying reasonably long job tenures. EwL is therefore not trivial, but rather a common and sustained feature of many young people's lives. The significance of EwL for informing values, expectations and shaping inequalities in young people's working present and future is therefore critically underplayed by transitions research.

Where researchers attend to EwL, the tendency has been to adopt a binary view centring on how it improves or undermines ‘employability’. On the one hand, some student work is seen as ‘CV‐enhancing’ (Howieson et al. 2010). Paid and unpaid internships (Leonard et al. 2016) are badged as preparing students ‐ albeit unequally ‐ for graduate employment (Allen et al. 2013; Lowe and Gayle 2007; Raby et al. 2018; Toft and Friedman 2021). On the other hand, paid work (particularly term‐time work) is regarded as a distraction or detrimental to academic outcomes, future employability and wellbeing (Broadbridge and Swanson 2005; Curtis and Shani 2002; Sanders, n.d.). The latter framing increasingly dominates national media, where students are depicted as ‘cash‐strapped’ and forced to choose between lectures and employment (Skopeliti 2023). Among these stories some positive discourses of EwL exist. For example, as universities work to embed work placements within university courses (Clarke 2018), the growth of the ‘student side hustle’ is lauded as entrepreneurial, and actively promoted by some universities (Allen and Finn 2023). The result of this ‘binary’ thinking (Roberts 2011) is that working while studying is under‐theorised and only partially addressed within both the youth transitions and employability paradigms.

A third conceptual approach frames EwL as ‘anticipatory socialization’ or ‘preparation for a “precarious life”’ (Billett and Ovens 2007; Rydzik and Kissoon 2022; Taylor 2022). Early experiences of EwL, such as hospitality work, are understood in these accounts as promoting an acceptance of harmful working environments. We build on this—currently less widely adopted—perspective by arguing for the need to conceive of EwL as work equivalent in significance to other types of work and recognise it as part of young people's working experiences. Doing so enables a recognition of the role of EwL in potentially influencing trajectories and choices over the working‐life‐course, including their gendered patterning.

### 2.2 Gendered Careers

Gendered outcomes, including vertical and horizontal segregation and gender pay gaps, are well documented and persistent (Blackburn et al. 2002; Goldin 2021). Partly this is explained by women and men being clustered in different occupations (gendered occupational segregation), with female‐dominant occupations typically offering lower pay and fewer opportunities for advancement (Leuze and Strauß 2016). In addition, women remain under‐represented at the higher levels of management and seniority (vertical segregation) (Cardador 2017; Hesmondhalgh and Baker 2015).

Individual‐level analyses of women's working lives and gendered disadvantage predominantly focus on significant life events namely childbearing and child‐rearing (commonly referred to as the ‘motherhood penalty’ e.g. L. Jones et al. 2023; Zamberlan and Barbieri 2023). These studies chart the ways in which the dual burden of care and work constrain women's employment choices, affecting both pay and occupational location. A small number of studies show, however, that the accumulation of gendered working life experiences may commence long before family formation (Combet and Oesch 2019). Research into gendered aspirations in different national contexts has tended to centre family‐formation focusing on how motherhood and work will be reconciled by young women (e.g. Gordon et al. 2005; McDonald et al. 2011; Patterson and Forbes 2012). Moreover, it has shown how for many women, ‘nearly all’ imagined future plans and predictions change (Thomson et al. 2004, 234).

Relevant to our study, evidence from the United States and Scotland has found occupational gender disparities at an early age, predating parenthood. This is evident in babysitting, some types of front‐line service work, and caregiving, where young women predominate, as well as in factory and delivery work, typically performed by young men (Besen‐Cassino 2018; Howieson et al. 2012; McKechnie et al. 2014). Alongside this, small‐scale studies in the United States, the Netherlands, and Canada identify gender pay gaps for young (even school‐age) workers, with disparities further exacerbated by race and social class (Besen‐Cassino 2018; Kooreman 2009; Taylor et al. 2020). As Besen‐Cassino (2018, 150) argues, the presence of such disparities challenges the notion that the youth labour market offers a ‘gender utopia’. To provide original contributions to this body of literature we use contemporary national data to analyse EwL in England, including how and whether this is gendered.

## 3 Data and Methods

Given the extant lack of knowledge about student employment and a desire to explore patterns with respect to employment rates, pay, hours, and occupations, as well as how these are gendered, we conducted secondary data analysis. In identifying data, an essential criterion was that students (and student workers) were a sufficiently sizeable group that we could develop within‐group comparisons.

### 3.1 Data

Our main analysis focuses on the Annual Population Survey (APS, Office for National Statistics 2024). This collects household and personal information spanning January to December each calendar year, meaning that it is up‐to‐date. Employment data in the APS is based on the Labour Force Survey (LFS), enhanced with a national boost sample, producing larger samples than the LFS, making it more suitable for analysis of relatively small sub‐groups. Data are collected from people aged 16 or older. Although not focused on students, APS includes large groups of students and asks for detailed information on their education and employment when applicable.

One advantage of the APS datasets is that they can come in different forms, and we conducted analyses with two of these forms: the APS 3‐year pooled datasets and the APS annual datasets. The APS 3‐year pooled datasets provide us with the most up‐to‐date data on large, boosted national samples and adequate occupational and income information for conducting cross‐sectional analysis. Among the over 340,000 observations in APS three‐year pooled dataset January 2021–December 2023 (referred to as APS Jan 21–Dec 23, Office for National Statistics 2024), there were 11,094 full‐time students and 3056 of them reported being in paid employment. Meanwhile, combining the annual datasets from 2005, 2010, 2015, 2019, and 2022 allowed us to compare students' employment over time for validation of the robustness of findings.^1^

### 3.2 Key Variables

Most analyses in this article use information on the young full‐time student samples from APS, for ages 16 to 22. This spans the period of compulsory education and the typical age profile for participation in further and higher education. In selected analyses we include full‐time students aged 23–29, encompassing the typical period for postgraduate education and/or education returners. The focus is full‐time students because most policy treats full‐time students as only and primarily students and because there are relatively few part‐time students in the 16–22 age‐group. We exclude apprentices because this is a separate category of activity involving more closely integrated work and study. Employment rates are calculated based on students who report their primary economic activity as ‘in employment’ in the survey.

Selected employment variables—such as working hours, hourly wages, and detailed occupational information—are used to answer specific research questions. Supporting Information S1: Appendix I provides definitions for these variables. In addition, to analyse the distribution of occupations and gender segregation among young student workers, we used three‐digit occupational variables and created a variable to classify the gender composition of each occupation *(occ_feature)*. This had four categories: female occupation, male occupation, integrated occupation, and small‐group occupation. Among occupations with at least three working students, we classified as ‘female occupation’ those with more than 66.6% female student workers, as ‘male occupation’ those with more than 66.6% male student workers, and as ‘integrated occupation’ those with a relatively balanced gender composition. Other empirical analyses apply a threshold of 70% to determine the gender‐dominant feature of occupations (Leuze and Strauß 2016). At the three‐digit level, occupations were dispersed and we decided a threshold at 66.6% to identify occupations with three people of different genders as gendered occupations. Occupations with fewer than three students are in the ‘small‐group occupations’ category. We are aware that 66.6% was an arbitrary threshold and altering the threshold potentially moves occupations between categories. For example, if we raised the threshold to 70%, ‘other skilled trades’,^2^ which had two female workers and one male worker in APS Jan 21–Dec 23, would change from a ‘female occupation’ to an ‘integrated occupation’. There was not however another threshold that neatly resolved such issues. Moreover, as discussed below, during analysis we took actions to control for occupations that moved between different groups at different times (e.g. moving from integrated to male). That said, we are cognisant of the limitations of this categorisation, and conservative about claims based on it.

There are several noteworthy decisions in variable choices. We excluded students who were in part‐time studies or forms of open educational programmes. The decision was made because part‐time students are empirically different to full‐time students. First, they are a relatively small group in this age‐group (*n* = 663 in APS Jan 21–Dec 23, 3.6% among 16–22‐year‐olds) with much higher employment rates and longer working hours. Critically their relationship to work is different; part‐time students have often returned to study *after* some labour‐market engagement and may even be studying for job‐related qualifications. Therefore, we decided not to combine full‐time and part‐time students in these analyses. The ways in which part‐time students combine work and study are, however, also insufficiently understood and should be explored in future analyses.

We also excluded students who reported self‐employment (0.6% of students) or being in government schemes (0.2%) for their rarity. We were not able to explore students' engagement in the gig economy because although the APS has recently produced experimental variables on students' engagement in the gig economy, these have not been made available for analysis in published datasets. Finally, we focused on young student workers' main jobs because only 0.8% of students reported a second job. We suspect, however, that the proportion of students engaged in more than one type of economic activity may be higher than this and that this activity is not always reported, especially where it is irregular (e.g. occasional agency shifts).

We note three limitations in this study. First, the data provide a snapshot. As such they are poor at capturing students in occasional or irregular work, or students who move in and out of jobs across different parts of the academic year. The second issue relates to income data quality, which is poor in these surveys (a perennial problem), with considerable missing information. Under‐reported income data was slightly more likely for 16–22‐year‐olds student workers (44% missing) than general workers older than 23‐year‐old (40%), perhaps because they had more variable hours which may make calculation more difficult and less precise. Finally, and related to the preceding two points, our findings should be read as relating only to formal and relatively regular student work, since these data rely on students and coders recognising and categorising activities as work for it to be captured. This means that informal, occasional, stigmatised or illegal work including babysitting, online‐selling, sex‐work or other less widely recognised earning activity will rarely be captured.

## 4 Results

To open up the ‘black box’ of student employment, this section presents a detailed descriptive picture of employment rates, hours, pay, and the occupational location of student workers, identifying gendered patterns in these.

Students typically worked fewer hours, earned less than other workers, including other young workers, and were much less likely to be self‐employed or work from home (Table 1). Almost all students worked in the private sector, and they were more likely than other young people to have variable working hours, suggesting that the work undertaken may be relatively insecure. Students and young workers (16–22) have relatively fewer years spent in the labour market, so it is unsurprising that their average current job tenure is lower than other workers. Given this age profile, the first notable finding is that 43% of student workers had employment tenure of more than a year. Such tenure suggests employment is relatively stable for a sizeable portion. Student workers were slightly less likely to identify as White, more likely to identify as Black or of Mixed ethnicity, and slightly more likely to be born in the UK than older workers, but these differences were relatively small.

**TABLE 1:** Workers^3^ in Annual Population Survey dataset, 2021 Jan–2023 Dec.

	Full‐time student workers (16–22) (*n* = 2950)	Non‐student young workers (16–22) (*n* = 4337)	All workers (16+) (*n* = 152,540)
Workers as per cent of populations (%)	26.6	57.3	53.2
Working hours (mean)	14.7	30.8	31.9
(sd)	(11.4)	(14.3)	(16.5)
Hourly wages (mean)	9.2	10.1	18.0
(sd)	(5.0)	(4.2)	(11.3)
Self‐employed (%)	2.2	5.4	14.4
Working from home (%)	4.2	8.7	28.4
Private sector (%)	92.7	88.0	74.5
Working hours vary (%)	33.6	26.4	31.5
Tenure of current job (%)			
< 3 months	20.2	14.5	3.8
3–6 months	16.2	13.9	4.0
6–12 months	21.8	18.0	6.4
1–2 years	21.5	22.2	9.4
2–5 years	19.6	28.7	20.1
5–10 years	2.0	2.6	17.6
10+ yrs	0.0	0.1	38.8
Race/ethnicity (%)			
White	85.6	91.3	89.1
Mixed_multiple	3.6	2.2	1.1
Asian/AsianBritish/Chinese	6.2	3.8	6.0
Black/African/Caribbean/BlackBritish	3.3	2.0	2.4
Arab and other	1.2	0.7	1.3
Not born in UK (%)	8.3	7.4	14.5

### 4.1 Student Workers' Employment Rates

Young women were more likely to work while studying than young men. Female students aged 16–22 were a third more likely to work than male, with rates of 31.4 versus 23.8 (see Table 2). We used other data sources other than the APS, which also confirmed this gender gap. For instance, respondents to the COSMO Wave One survey were younger (typically 16–17 years‐old) with lower employment rates. Yet they displayed an even more striking gender disparity, with 50 per cent more female students participating in paid employment than male students. Interestingly, students identifying as non‐binary reported even higher rates of employment. In our final data set, Next Steps, which includes students 16–21 years‐old, rates of work were slightly lower than we found in the APS (perhaps because of the slightly younger age group), but again female students were more about 50% more likely than their male counterparts to work. These gender differences in employment rates are statistically significant in all three datasets. To provide sense of the scale of these differences we show that in the COSMO data the gender gap is greater than the disparity between students in public and private schools (school type differences also lacked statistical significance in this analysis). Gender therefore appears to be a key variable in shaping employment rates amongst students.

**TABLE 2:** Employment rates among young students. APS 3‐year pooled Jan 2021–Dec 2023, COSMO Wave 1, and Next Steps 2004–2015.

	Per cent of 16–22 full‐time working students, APS Jan 2021–Dec 2023	Per cent of Y12 (sixth form/college) working students, COSMO W1 2021/22^4^	Per cent of 16–21 full‐time working students, next steps wave 4–7 2007–2010^5^
Gender^a^			
Male	23.8	9.9	19.6
	(*n* = 1298)	(*n* = 5850)	(*n* = 20,162)
Female	31.4	15.5	28.4
	(*n* = 1758)	(*n* = 6666)	(*n* = 20,337)
Non‐binary		17.3	
		(*n* = 228)	
School type			
State		13.0	
		(*n* = 13112)	
Private		8.6	
		(*n* = 674)	

a All the comparisons by gender are statistically significant.

The percentage of students identified as engaged in work in either APS, COSMO, or Next Steps is lower than that reported in some previous studies. This is partly because we focus here on snapshots of respondents' *current* work, whereas other studies report on whether students undertake any paid work across the past academic year (NatCen Social Research & Institute for Employment Studies 2023) or a 2‐year period (Office for National Statistics 2017). Students with irregular jobs or going in and out of employment may not be identified by a snapshot. Additionally, a few studies focused specifically on young people or students have included additional measures to capture types of informal work that are not captured by the APS. There is not, however, an a priori reason why the much greater female engagement in student work (seen in Table 2) would be altered if the data accounted better for either informal or irregular employment.

To further check the robustness of our finding of a large gender disparity in employment rates, and to explore how this gendering is patterned by age we examined multiple waves of APS data over the past 2 decades, splitting the data by age‐group.^6^

**FIGURE 1:**
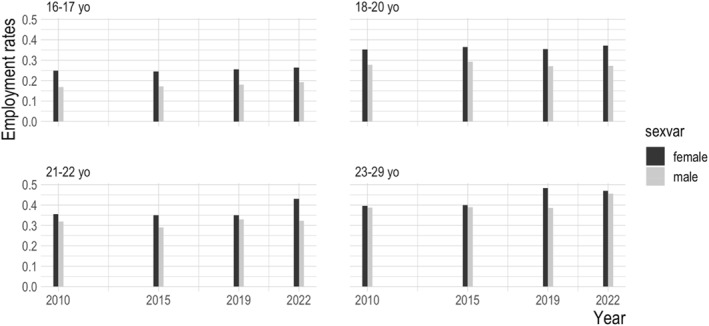
Employment rates among male and female full‐time students, APS Jan–Dec 2010, 2015, 2019, 2022, by age‐group.

Figure 1 shows higher employment rates among female than male students across all age groups and every time point. These differences were statistically significant for the two youngest age groups (16–17‐ and 18–20‐year‐olds) at every time‐point examined. They were significant for 21–22‐year‐olds at almost every time‐point (except 2019). Among the oldest (and smallest) group of students (23–29‐year‐olds) the difference in rates of work between male and female students was typically smaller and only statistically significant in 2019. Students of this age are, however, more diverse and differentiated, many returning to study after or alongside paid work. It is, thus, a persistent finding that female students between 16 and 22 (the usual ages for sixth form, college and undergraduate education) are significantly more likely than men to be working alongside their studies, and that older students are more likely to engage in EwL. Lastly, there is corroboration for other studies (cited above) that show the percentage of students engaged in paid work has increased in recent years, especially for 21–22‐ and 23–29‐year‐old students.

### 4.2 Student Workers' Hours

**FIGURE 2:**
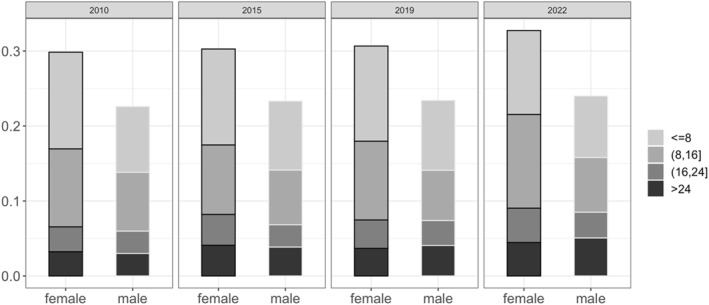
Working hours distribution among student workers, by gender, APS 2010, 2015, 2019, & 2022, 16–22 year‐old full‐time students.

We examine students' working hours distribution in Figure 2, breaking it down by gender. Long hours were rare for both genders, with only about 5% of full‐time students working longer than 24 h per week and another 5% working between 16 and 24 h. The proportion of students engaging in long hours increased a little, especially by 2022. However, most full‐time students engaged in EwL worked relatively few hours. Similar numbers of male and female students were working over 16 h per week, but there were more female than male students engaging in low‐hours EwL (up to 16 h per week).

Although most students worked short hours, many worked across multiple days of the week. Among those who reported which days in the reference week they had worked, 17.4% reported undertaking work on just one day. More common was to work across 2 days (26.2%) or 3 days (16.8%). There were however, 11.8% of students who reported that they had undertaken paid work across five or more days. In sum, in just under half of cases, EwL meant working across at least 3 days of the week, meaning it stretched across a significant proportion of their week (see Supporting Information S1: Appendix II).

### 4.3 Student Workers' Pay

**FIGURE 3:**
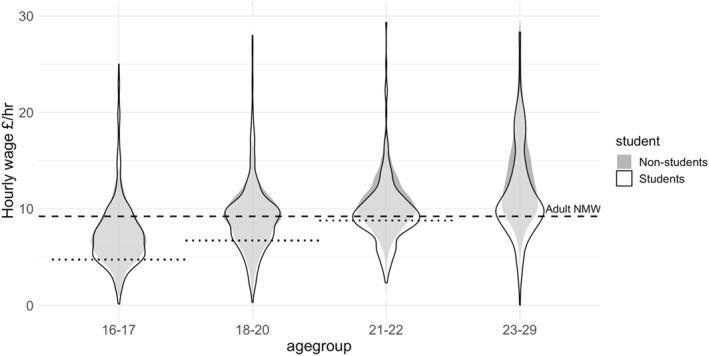
Hourly wage distribution among student and non‐student workers, APS Jan 2021–Dec 2023.^7^

We used the APS Jan 21‐ Dec 23 data to analyse hourly pay for student workers and non‐student young workers. Figure 3 includes four violin plots, one for each age group. These represent the density—or proportion of student workers—at each earning point (in GBP). Each plot includes a shaded area describing the spread of pay for non‐student workers and an outlined area showing the spread of pay for student workers. The more that these spreads overlap the closer the distribution of pay for the two groups. There is a dashed horizontal line showing the average adult minimum wage (NMW) across the period. There are also shorter dashed lines to indicate age‐differentiated NMW rates. We include these to relate actual pay to legal minimums. Because the NMW increases annually, the lines represent average NMWs from 2021 to 2023.^8^ That means that any particular case just below may actually fall within the NMW for the time point of the interview and vice versa where a case is just above. Across the period, however, the proportion of cases above/below should approximate the proportion of workers being paid more/less than the NMW. Large parts of each plot fall below the relevant age‐differentiated NMW line, suggesting a large minority of respondents in each age‐group are paid *below* the legally mandated NMW. While that means, conversely, that most students are paid above the relevant age‐differentiated NMW, only a small minority of 16–18‐year‐olds, a larger minority of 19–20‐year‐olds and just over 50 per cent of 21–22‐year‐olds are paid at or over the adult NMW. In the first two cases the majority of the student plot falls well below the heavy dashed line. While most 23–29‐year‐old students are paid over this (formally adults under this legislation), not all are. In other words, most employers take advantage of (lower) age‐differentiated NMW rates to pay student workers less than they would older workers. In addition, a significant minority of students within each age group earn less than the (already low) age‐specific legal minimum. To check that this finding was not simply the result of respondents in our dataset being interviewed at different time points between January 2021 and December 2023, we used respondent interview month to more precisely investigate the relationship between young people's wages and (changing) age‐specific and adult national minimum wage levels (see Supporting Information S1: Appendix III). This analysis confirmed that across every period significant minorities of student workers earnt below age‐specific NMWs and a much larger proportion were paid below the adult NMW.

Comparing the pay profiles of student workers and non‐student workers across these age groups, we can also see that ‘studentness’ operates as a penalty for some groups of young workers. Specifically, these plots reveal a small but statistically significant student pay penalty for older students (students aged 21–22 and 23–29). For these groups the student plot is wider at the bottom (lower pay rates) and the non‐student plot is wider at higher pay‐points. More older students are therefore in low‐pay, below‐NMW work than non‐student workers of the same age. However, for the very youngest students (16–17) who earn the least there is no consistent difference between student and non‐student earnings (Figure 3). Indeed, the data suggest that in some time periods non‐students were more likely to earn below age‐specific minimum wages (see Supporting Information S1: Appendix III). However, non‐student 16–17‐year‐old workers are rare, and relevant cell sizes small.

Turning to gender, in most age groups and years, average male wages were slightly higher than average female wages. However, this was not always true, and there were no statistically significant gender wage gaps among student or non‐student workers for any of age groups 16–17, 18–20, or 21–22 (with one exception—a small female pay advantage in 2015 for 21–22‐year‐old non‐students) (see Supporting Information S1: Appendix IV). This suggests a high level of gender equality and limited early pay gaps. Kooreman's (2009) research in the Netherlands found a gender gap in both total and hourly pay, although this included more informal, typically very poorly paid feminised types of work (especially babysitting), which the data presented here do not. Future analyses of younger workers would, therefore, benefit from collecting data on less formal types of work.

### 4.4 Student Workers' Occupations

Besen‐Cassino (2018) and Kooreman (2009) identify specific occupations (babysitting archetypically) as relevant to the development of early gender inequalities. Given this and a well‐documented relationship between pay inequalities in later life and occupational segregation (Bloksgaard 2011; Fritsch et al. 2022), we explored student workers' occupations and whether and how these are gendered.

Table 3 presents the 12 largest non‐professional occupations^11^ for student workers showing whether the occupation is largely integrated (approximately equal numbers of male and female workers), male or female dominated; the weighted mean hourly wage in each occupation; and the gender pay gap.^12^ Higher positive numbers reflect a larger men's pay advantage. Lower negative numbers mean a female advantage. Numbers close to zero mean there is little to no gender pay‐gap for students in that occupation. These 12 occupations make up 82% of non‐professional work among full‐time students. Most of that (62%) is accounted for by just two occupational groups, ‘other elementary services occupations’ and ‘sales assistants and retail cashiers.’ These two student occupations are integrated, but poorly paid (both are among the four lowest paid occupations here) and have very small gender pay gaps.

**TABLE 3:** Twelve largest non‐professional occupation groups among student workers APS Jan 2021–Dec 2023, 16–22 full‐time student workers.

Occupation names	Occupational gendering	Total	N income reports	Weighted mean hourly wage (£/hr, deflated with 2021 as base)^9^	Gender wage gap (male advantage over female %)^10^
Other elementary services: e.g. bar staff, coffee shop workers, waiter and waitresses	Integrated	1095	636	7.0	−2.2
Sales assistants and retail cashiers	Integrated	711	401	7.8	1.3
Caring personal services	Female	89	47	9.1	−27.2
Customer service occupations	Integrated	87	41	8.2	−19.9
Sports and fitness occupations	Integrated	79	38	7.4	45.7
Leisure and travel services	Integrated	61	37	8.3	26.9
Elementary cleaning occupations	Integrated	61	31	8.0	33.4
Elementary storage occupations	Male	53	29	10.3	−20.6
Elementary sales occupations	Integrated	51	29	8.4	34.8
Teaching and childcare support occupations	Female	49	26	6.9	−2.2
Other administrative occupations: e.g., sales administrators, data entry administrators	Integrated	38	23	10.0	45.2
Elementary administration occupations: e.g., postal workers, call centre agents	Male	37	20	5.5	53.5

**FIGURE 4:**
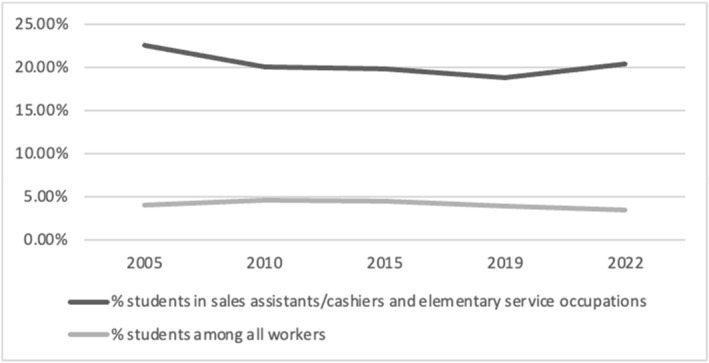
Share of students in the general workforce VS. sales/service sector. APS 2005, 2010, 2015, 2019, & 2022, 16–29 year‐old full‐time students.

Although students are under 5% of the national workforce, they account for over 20% of all sales assistants, retail cashiers and elementary service workers (Figure 4). LFS OD2022 data enabled us to identify that most student workers in these two occupational groups work in the following locations: restaurants and mobile food service activities; beverage serving activities (such as bars and cafes); retail sale in non‐specialised stores with food, beverages or tobacco predominating (such as supermarkets); and hotels and similar accommodations. Although unsurprising, this is a useful reminder of how heavily businesses in these sectors rely on student labour and as such, how consequential (low) wages in these sectors are for student income and, relatedly, wellbeing.

### 4.5 Student Workers' Occupational Gendering

**FIGURE 5:**
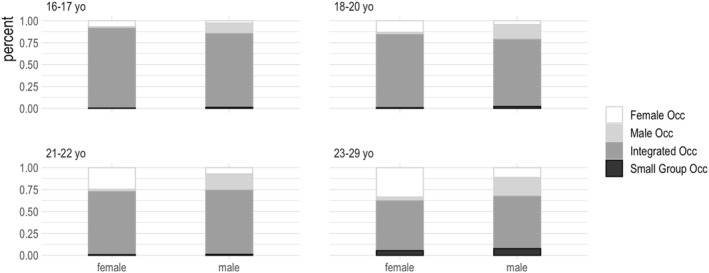
Distribution of full‐time student workers in occupation groups, APS Jan 2021–Dec 2023, by age‐group.

Figure 5 shows the distribution of male and female students across female, male, integrated and small group occupations within each of the four age groups. Clearly, most students worked in integrated occupations, including almost all the youngest student workers. At older ages, more student workers moved into gender‐dominant occupations. We also see growth in the fractions of students in small group occupations, signalling older students' involvement in increasingly diverse (potentially less ‘studenty’) types of work.

**FIGURE 6:**
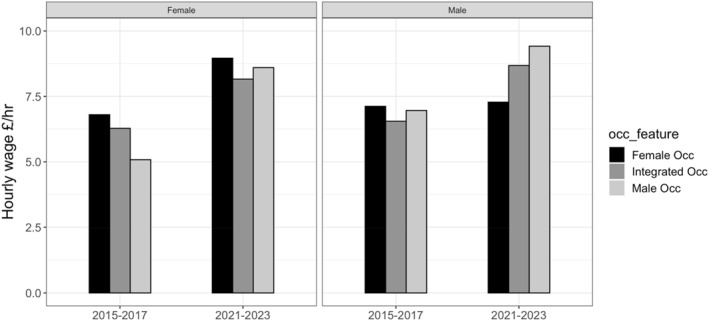
Pay gaps between occupation groups, by gender. APS Jan 15–Dec 17 VS. Jan 21–Dec 23, 16–22‐year‐old full‐time student workers.

We examined whether gendered occupations produce wage advantages either for all students in the occupation, or for either men or women. Figure 6 presents some evidence that more gendered occupations produced pay advantages for dominant‐gender workers. Female student workers had the highest earnings in female‐dominant occupations in both the 2015–2017 and 2021–2023 data, and male workers had the highest earnings in male‐dominant occupations in 2021–2023. Given relatively small cells (especially for women/men in gender‐opposite sectors) we only found a significant wage difference between occupational types for women in 2015–2017 (*p* < 0.01**).

Meanwhile, student workers experienced pay disadvantages when working in integrated occupations or in occupations dominated by the other gender. In 2015–2017, male workers were paid worst in integrated occupations and female workers had the worst pay disadvantage in male occupations. In more recent years 2021–2023, men working in female occupations were the lowest earners, while women were worst off in integrated occupations. Focusing only on workers in integrated occupations (which accounts for 83% of workers in each period), we found a small but statistically significant male wage advantage over female workers (*p* < 0.05**) in 2015–2017. Men retained an advantage in 2021–23, but it was not significant.

We therefore find that relative pay equality is primarily produced by the concentration of the vast majority of students in poorly‐paid gender‐integrated occupations. As student workers get older, however, they are more likely to move into gender‐segregated work. As they do, evidence shows that they experience pay advantages for gender‐matching, suggesting an early labour‐market incentive for occupational gender segregation.

### 4.6 Summary

This article uses national data to open up the ‘black box’ of work undertaken by young full‐time student workers in England. In this section we summarise key findings.

Our first and most striking finding is that young women are more likely than men to work while studying. This finding persisted across age groups and over time. It is especially pronounced among school‐aged workers (under 18s). Second, we find generalised poor pay, with most student workers paid below the adult NMW and a sizeable minority paid below lower age‐defined legal minimums. Given the finding that for many students these are ongoing jobs, these findings show that students may experience extended periods of very poorly compensated employment.

Third, we identify a ‘studentness penalty’ with student workers aged 21–22 and 23–29 on average earning less than non‐student workers of equivalent age. This may relate to specialisation (or students' lack thereof), or that students are seen by employers as an especially exploitable workforce, even compared to their poorly‐paid peers, or it may be that students themselves are willing (even if not happy) to tolerate poorer wages than other young people.

Fourth, we find relative gender pay ‘equality’ but show that this is related to the concentration of both male and female students in two very poorly paid occupations. Most employers take advantage of (lower) age‐differentiated NMW rates to pay young student workers less than they would older workers. As such what we find is not a gender‐equal utopia, but rather equally low pay for the large numbers of students engaged in such work. As such we advance studies that have highlighted youth disadvantage and underemployment (Churchill and Khan 2021), showing that the disadvantages of student work are unequally distributed among men and women students.

Fifth, we find that students move into increasingly gendered occupations at older ages. Moreover, we find some evidence of pay advantages to working in dominant‐gender occupations. These findings are not significant at all time points but are at a minimum suggestive of potential incentives for students to move to more gender‐typical occupations. As such this suggests that decisions producing gendered working‐life‐course trajectories and widespread occupational segregation in later working lives may be rooted in work undertaken by very young people, including earning while learning.

Finally, in sum, encapsulating much of the above, our analysis suggests that waged labour is an important, time‐consuming and, for many, enduring part of student life, especially for young women.

## 5 Conclusion

Student work is widespread, but poorly paid and women are more likely than men to engage in this work. This raises new questions that warrant further attention. First, what is motivating young women and non‐binary students into earlier labour force entry than young men? Second, in a longer view, how do these early experiences of work relate to later working life trajectories and outcomes? We return to these questions below.

The research presented is both empirically and theoretically novel, as student work is so under‐studied that there has been virtually no prior analysis or theorisation of gender differences in employment among students. Solely based on the quantitative data presented, it is not possible to evidence why women students report higher rates of employment, but we offer some potential explanations. As we show, student workers are predominantly located in retail and hospitality, and other research suggests that young workers, but particularly women, are attractive to employers in these sectors due to the kinds of emotional and aesthetic labour demanded (Besen‐Cassino 2018; Coffey et al. 2018). As Farrugia (2021, 380) argues ‘middle class young women—often students—are best equipped with the kinds of classed and gendered dispositions required to successfully perform this labour’. Thus, gender norms mean that young female students may be seen as particularly compliant and well‐behaved workers, as compared to their male peers or early school leavers. Employer demand may therefore be a factor. Push factors may also play a role, not least expectations placed on young women to be self‐sufficient and independent. The Student Income and Expenditure survey of higher education students in England shows that female students receive less income from families than men (NatCen Social Research 2018), meaning that higher employment rates might be explained by young women's lesser access to economic resources or family support.

Exploring the implications of students' early experiences of paid work on later outcomes and working trajectories is a key component of future research agendas. The research here suggests that the extent to which early employment sets expectations, establishes workplace discipline and shapes (gendered) occupational preferences requires further examination. In light of our findings, we therefore reject extant frameworks for theorising student work—either those that treat work and education as two separate time‐ordered and typically hostile spheres (as is typical of the transition literature), or those in which students are only acknowledged as ‘real workers’ after completion or exit from education (as is found in the employability literature). We suggest instead that we can better understand young lives and identities by appreciating the economic and social significance of typically part‐time work undertaken alongside education. Such a theorisation necessitates an expansive conceptualisation of young people as simultaneously workers *and* students. We propose that a ‘working‐life‐course’ approach, rooted in life‐course analysis (Ford et al. 2021; Moen and Han 2001), may be a fruitful way forward. One aspect of a working‐life course approach would be to understand how work and study interact contemporaneously in ways that produce formative gendering of work, in both the present and the future.

Both our empirical analysis and reconceptualisation have policy relevance as these reveal the significant contribution of student workers to the economy and therefore the importance of recognising them as a key group within the labour force. Such recognition has practical implications in that it necessitates such ‘student workers’ be treated as real workers whose rights at work, pay, and conditions are as deserving of protection as that of others. As such, we suggest that labour protections must extend to students. Given that women disproportionately undertake student work, the extension of such rights and protections would be a key step‐change in bringing about greater gender equality in the labour market and in the workplace.

## Supporting information

Supporting Information S1

## Data Availability

The data that support the findings of this study are openly available in datasets from APS Surveys at https://ukdataservice.ac.uk/, reference number 9291, 5395, 6809, 7928, 8632.

## Endnotes

^1^
LFS survey was largely impacted by COVID19 in response rate (See: Helme 2022, *LFS & APS User Up‐date*, UKDS Annual LFS/APS User Conference 2022). Given concerns about data collection during that period, and changes in work and education practices over that time, we omitted 2020 and 2021 as peak pandemic periods while spreading data points relatively evenly to ensure historical comparison. Unavoidably there are 2021 data included in the APS Jan 2021–Dec 23 dataset. We therefore sometimes triangulate these data with 2015–2017 APS data.

^2^
Including occupations like glass and ceramics makers, decorators and finishers, furniture makers, florists, etc.

^3^
Workers in the three sample groups all exclude apprentices.

^4^
COSMO Wave One: we use the first wave of the COSMO Survey (COVID Social Mobility & Opportunities Study, Anders et al. 2023), which follows a nationally representative sample of individuals who were in Year 11 during the 2020–21 academic year in England. The sample set extracted from COSMO Wave One comprises 12,744 students who entered Year 12 (sixth form/college, age 16–17) in 2021–22 when surveyed. COSMO offers researchers a unique opportunity to focus on young students who encountered the pandemic during a critical transitional life‐course phase but for our purposes it is also a valuable comparative counterpart to APS 2022 since both were conducted at a similar time and COSMO Wave One comprises a substantial student sample.

^5^
Next Steps (Centre for Longitudinal Studies 2024) is a national longitudinal panel study that follows a cohort of student from year 2001 when there were between 13 and 14 years old. We pooled together the records from wave four to seven, when students were between 16 and 21, and calculate the employment rates based on total person‐waves working.

^6^
There were missing weights for APS 2005 and so these data are omitted here.

^7^
Both student and non‐student wage distributions exclude apprentices and workers earning more than £30/hr (0.9% student and 2.5% non‐student workers).

^8^
The adult NMW was renamed the National Living Wage in 2016, but its legal status remains as a minimum. All minimum wage lines in Figure 3 are averages of the April 2021 to March 2022 and April 2022 to March 2023 rates https://www.gov.uk/national‐minimum‐wage‐rates.

^9^
Income has been deflated to 2021 values using Bank of England inflation figures for 2021–2023.

^10^
Gender gap in wages is measured as (weighted average male pay ‐ weighted average female pay)/weighted average female pay. Positive value means male pay advantage and negative value means female pay advantage.

^11^
We exclude professional occupations (six per cent among all student workers) which typically require educational credentials and may be understood as a more direct outcome of education.

^12^
The gender pay gaps in Table 3 were calculated as follows: for each occupation, paygap in % = (avg_pay_male − avg_pay_female)/avg_pay_female.
