# Supplementary material for: Equally Bad, Unevenly Distributed: Gender and the ‘Black Box’ of Student Employment
Source: Br J Sociol. 2025 May 4;76(4):828–40. doi: 10.1111/1468-4446.13210 (PMC12412076; doi:10.1111/1468-4446.13210)
Supplement: Supplementary file 1 — Supporting Information S1 [file BJOS-76-828-s001.docx]

**Appendix I**

**Table AI. Definitions/constructions of key variables**

| **Variables** | **Definitions/constructions** | **Notes** |
| --- | --- | --- |
| Fulltime student | We classified respondents as full-time students if they were: (1) currently attending school on a full-time basis, (2) enrolled in a sandwich course, or (3) pursuing full-time education at a university or college (variable *CURED8*). | Other (excluded) categories of types of course are: (4) Part time at school, (5) Training in nursing, (6) Part time at university or college, (7) Open college, (8) Open university, (9) Other correspondence course, (10) Course not stated, (11) Enrolled, but not attending, (12) Enrolled but not stated if attending, and (13) Not enrolled on course. |
| Employed | We classified respondents as ‘employed’ if they selected the category ‘in employment’ under the variable *ILODEFR – basic economic activity*. |  |
| Working hour | We used ‘total actual hours worked in main job in reference week (including overtime)’ (variable *TTACHR*) to identify students’ weekly working hours. | Actual working hours are generally shorter than usual working hours but, because they relate to the most recent week, better reflect experience. Actual hours may, however, undercount hours where someone has not been in work in the reference week (because they were sick, on leave or other). |
| Hourly wage | We compared individuals’ income levels using the variable ‘HOURPAY’, which is derived average gross hourly pay amounts based on reported income and weekly working hours. |  |
| Occupation | We used three-digit occupational variables in the APS data (SC20MMN and SC10MMN). | An advantage of APS data is that it provides detailed information on respondents’ occupations. |

**APPENDIX II.**

**Table AII: Number of days reported scheduled working in the reference week**

**APS Jan 2021 – Dec 2023, 16 – 22 fulltime students**

| Days | 0 | 1 | 2 | 3 | 4 | 5 | 6 | 7 | Total |
| --- | --- | --- | --- | --- | --- | --- | --- | --- | --- |
| n | 635 | 532 | 801 | 513 | 215 | 303 | 36 | 21 | 3056 |
| % | 20.8 | 17.4 | 26.2 | 16.8 | 7.0 | 9.9 | 1.2 | 0.7 |  |

**APPENDIX III.**

**Table AIII: Proportions of poorly paid young workers**

|  | **Jan21-Mar21** | |  | **Apr21-Mar22** | |
| --- | --- | --- | --- | --- | --- |
| Age-group | Students | Non-students | Age-group | Students | Non-students |
|  | (n=196) | (n=1206) |  | (n=1207) | (n=6375) |
| *Weighted proportion of workers below national* ***age-specific*** *minimum wage levels* | | | | | |
| 16-17 | 18.3 | 54.6 | 16-17 | 17.1 | 8.2 |
| 18-20 | 36.0 | 25.5 | 18-20 | 29.6 | 18.3 |
| 21-24 | 65.0 | 22.5 | 21-22 | 28.9 | 25.6 |
| 25-29 | 55.9 | 14.4 | 23-29 | 35.9 | 13.7 |
| Apprentices | 0.0 | 10.4 | Apprentices | 14.8 | 7.6 |
| *Weighted proportion of workers below national* ***adult*** *minimum wage levels* | | | | | |
| 16-17 | 65.4 | 100.0 | 16-17 | 76.1 | 84.0 |
| 18-20 | 60.3 | 54.3 | 18-20 | 62.6 | 56.5 |
| 21-24 | 66.0 | 32.4 | 21-22 | 47.2 | 32.9 |
| 25-29 | 55.9 | 14.4 | 23-29 | 35.9 | 13.7 |
| Apprentices | 47.8 | 56.4 | Apprentices | 43.6 | 51.2 |
|  |  |  |  |  |  |
|  | **Apr22-Mar23** | |  | **Apr23-Dec23** | |
| Age-group | Students | Non-students |  | Students | Non-students |
|  | (n=1236) | (n=6801) |  | (n=234) | (n=1358) |
| *Weighted proportion of workers below national* ***age-specific*** *minimum wage levels* | | | | | |
| 16-17 | 21.3 | 10.5 |  | 21.5 | 9.9 |
| 18-20 | 21.0 | 17.6 |  | 47.1 | 26.5 |
| 21-22 | 38.6 | 24.5 |  | 88.8 | 46.5 |
| 23-29 | 34.7 | 13.4 |  | 40.6 | 17.4 |
| Apprentices | 9.8 | 8.3 |  | 4.0 | 8.1 |
| *Weighted proportion of workers below national* ***adult*** *minimum wage levels* | | | | | |
| 16-17 | 78.4 | 81.3 |  | 89.0 | 100.0 |
| 18-20 | 52.4 | 57.5 |  | 84.1 | 74.3 |
| 21-22 | 45.7 | 32.5 |  | 88.8 | 49.1 |
| 23-29 | 34.7 | 13.4 |  | 40.6 | 17.4 |
| Apprentices | 37.2 | 46.2 |  | 83.7 | 45.1 |

**APPENDIX IV.**

**Table AIV: Gender Wage Gaps by age groups.**

**APS Jan – Dec 2015, 2019, 2022, 16-22 fulltime students**

| Year | Agegroup | Avg_pay_male | Avg_pay_female | Pay gap |  |
| --- | --- | --- | --- | --- | --- |
| 2015 | 16-17 | 5.27 | 5.80 | -0.53 |  |
| 2015 | 18-20 | 6.61 | 6.55 | 0.06 |  |
| 2015 | 21-22 | 6.76 | 7.00 | -0.24 |  |
| 2019 | 16-17 | 6.60 | 6.49 | 0.11 |  |
| 2019 | 18-20 | 7.37 | 8.13 | -0.76 |  |
| 2019 | 21-22 | 9.00 | 9.01 | -0.01 |  |
| 2022 | 16-17 | 7.54 | 7.50 | 0.04 |  |
| 2022 | 18-20 | 9.67 | 8.96 | 0.71 |  |
| 2022 | 21-22 | 9.99 | 9.69 | 0.3 |  |

**APS Jan – Dec 2015, 2019, 2022, 16-22 non-students**

| Year | Agegroup | Avg_pay_male | Avg_pay_female | Pay gap |  |
| --- | --- | --- | --- | --- | --- |
| 2015 | 16-17 | 4.55 | 4.37 | 0.18 |  |
| 2015 | 18-20 | 6.64 | 6.40 | 0.24 |  |
| 2015 | 21-22 | 8.16 | 7.93 | 0.23* |  |
| 2019 | 16-17 | 5.62 | 5.65 | -0.03 |  |
| 2019 | 18-20 | 7.58 | 7.81 | -0.23 |  |
| 2019 | 21-22 | 9.73 | 9.37 | 0.36 |  |
| 2022 | 16-17 | 6.42 | 7.16 | -0.76 |  |
| 2022 | 18-20 | 8.97 | 8.99 | -0.02 |  |
| 2022 | 21-22 | 11.1 | 11.3 | -0.2 |  |
